# Single-Session Surgical Intervention with Resection of a Primary Cranial Osteosarcoma and Cranioplasty using a 3D-Printed Craniotomy Template and Cranioplasty Molds

**DOI:** 10.1055/a-2508-0868

**Published:** 2025-01-23

**Authors:** Sven Duda, Philipp Ivanyi, Mohamed Omar, Christian Hartmann, Eugen Musienko, Heinrich Wessling

**Affiliations:** 1 Department of Neurosurgery, Hospital of the German Armed Forces, Westerstede, Germany; 2 Department of Hematology, Hemostasis, Oncology and Stem Cell Transplantation, Hannover Medical School, Hannover, Germany; 3 Department of Traumatology and Orthopedic Surgery, Hannover Medical School, Hannover, Germany; 4 Department of Neuropathology, Hannover Medical School, Hannover, Germany; 5 Department of Mechanical Engineering, Laboratory for Manufacturing Technology, Helmut Schmidt University/University of the German Armed Forces, Hamburg, Germany

**Keywords:** cranial osteosarcoma, 3D printing, EURAMOS, craniofacial osteosarcoma

## Abstract

Although osteosarcomas are the most frequent primary malignant bone tumors, the primary cranial manifestation of this condition is very rare with only a limited number of cases presented in the literature.

We present the case of a 20-year-old male patient who underwent single-session surgical intervention for resection of right frontal osteosarcoma with a tailor-made craniotomy and cranioplasty using virtually designed 3D-printed templates and molds. Subsequently, the patient was treated according to the EURAMOS protocol and received adjuvant systemic chemotherapy.

At 18-month follow-up, the patient was clinically asymptomatic, and both the magnetic resonance imaging scan of the head and the staging computed tomography showed no signs of tumor recurrence or metastases. The case presented shows that the use of 3D-printed molds facilitate a safe preoperative planning of the resection area and a single-session surgery including a custom-made cranioplasty responding to the highest esthetical standards.

## Introduction

With an age-dependent incidence of 2 to 3/million/year in the general population, osteosarcomas stand as the most frequent primary malignant bone tumors. Nevertheless, primary osteosarcoma of the skull is a rare condition, accounting for less than 2% of all osteosarcomas. 1 The age distribution follows a bimodal pattern, with the first peak occurring between the ages of 10 and 14 years and a second peak among individuals over the age of 65. 2 Males are affected 1.4 times more often than females. 3 However, cranial osteosarcomas have been reported at a median age of 38 years and show only a slight tendency toward male predominance. 4 Osteosarcomas predominantly arise from mesenchymal cells of the long tubular bones, usually near the metaphyseal plate, with the femur, tibia, and humerus being the most common sites of tumor manifestation. 2 Following tumor resection and systemic chemotherapy, the overall 5-year survival rate is 68% in general and 51% in patients with cranial osteosarcoma. 2 4 In cases of cranial osteosarcomas, the presence of metastases at the time of initial diagnosis is uncommon and less frequent compared with osteosarcomas in other localizations, with rates 4, 7, and 18%, respectively. 4 Advanced age, metastatic disease, locally advanced disease, and tumor recurrence are factors associated with a poor outcome. 4 5

## Case Presentation

**Fig. 1:**
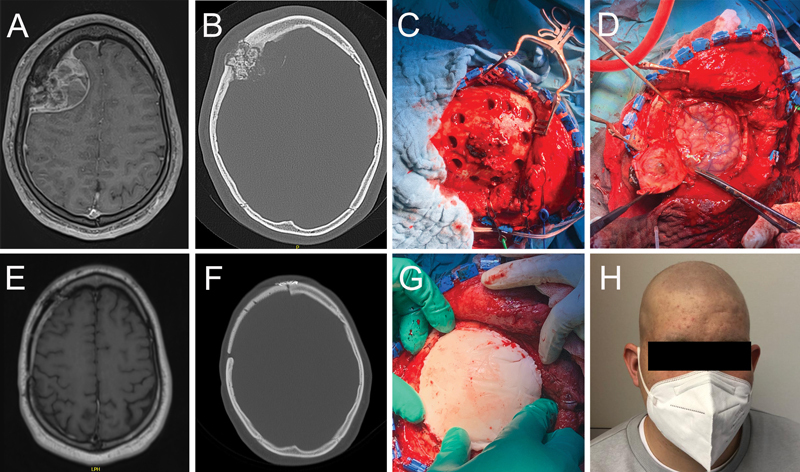
Case presentation. ( **A** ) Preoperative magnetic resonance imaging (MRI) scan, T1 contrast-enhanced. ( **B** ) Preoperative computed tomography (CT) scan. ( **C** ) Surgical site showing the outline of the osteoclastic craniotomy. ( **D** ) Surgical site highlighting the dural involvement. ( **E** ) Postoperative MRI, T1 contrast-enhanced. ( **F** ) Postoperative CT scan. ( **G** ) Positioning of the manufactured implant in situ. ( **H** ) Postoperative photography of the patient depicting the harmonic skull silhouette after a custom-made bone implant.

A 20-year-old man presented to our outpatient unit with a progressively painful swelling on the right side of his forehead. He had been aware of an irregularity on the right frontal surface for nearly 10 years by the time of his presentation. He began experiencing pain while playing soccer, especially when heading the ball, prompting a medical investigation. A head computed tomography (CT) scan revealed a cystic, osteolytic lesion with expansion both intra- and extracranial, measuring 40 × 39 × 46 mm. No edema of the adjacent brain tissue was visible, thus indicating a slow-growing tumor. This hypothesis was supported by a follow-up thin-slice CT scan taken 6 weeks later, which showed no enlargement of the lesion. Nonetheless, given the progression of symptoms, the patient was promptly scheduled for surgery. A month later, a magnetic resonance imaging (MRI) scan aimed at characterizing the lesion indicated an alarming size increase of the tumor, now measuring 64 × 54 × 47 mm leading to a significant mass effect and cerebral midline shift of almost 10 mm ( Fig. 1A ). Suspecting a malignant transformation, we planned for a gross total tumor resection, including a safety margin of at least 10 mm around the entire circumference. Further imaging including thorax and abdominal CT revealed no signs of metastatic disease. Our initial plan involved a single-session surgery that would involve tumor resection and simultaneous cranioplasty using a prefabricated implant. This approach aimed to prevent the typical temporary cosmetic deficit associated with the traditional two-step procedure including osteoclastic craniotomy followed by reconstruction. We ruled out a free-hand cranioplasty with polymethylmethacrylate (PMMA) due to cosmetic concerns. Despite the unexpected dynamics of the tumor's growth, we still deemed a single-session strategy as the most convenient way to address the patient's condition. This was especially important considering that in case of malignancy, additional treatments like chemotherapy could be delayed by a second surgery. Alternatively, the young patient would have to endure an extended period with a visibly disfiguring cranial defect. Thus, we were in need of an approach that would enable immediate tumor resection and cranioplasty in a single intervention.

### Surgical Procedure

**Fig. 2:**
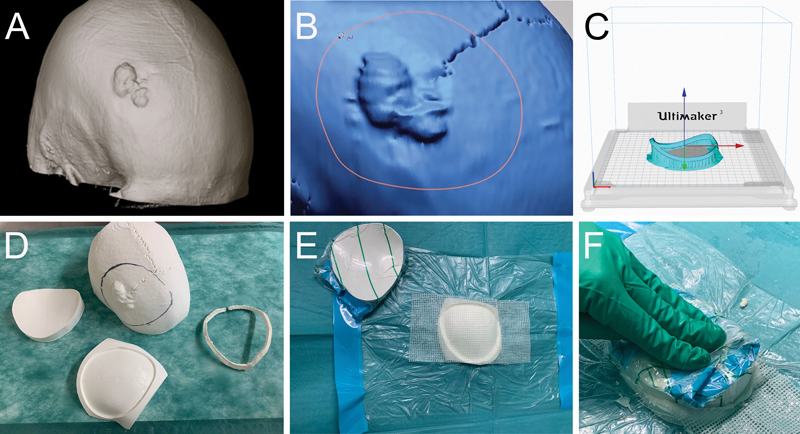
Manufacturing of the cranioplasty implant. ( **A** ) Exophytic growth of the tumor shown on a three-dimensional (3D) volume rendering of the cranial computed tomography scan. ( **B** ) Virtual planning of the craniotomy outline. ( **C** ) Exemplary picture of the 3D printing process of the craniotomy template. ( **D** ) Depiction of the 3D-printed craniotomy template, craniotomy molds, and a skull for preoperative testing. ( **E** ) Preparation of the cranioplasty molds using a sterile drape and fatty gauze. ( **F** ) Manufacturing of the tailored cranioplasty implant from polymethylmethacrylate using the patient-specific mold.

The patient was positioned supine with the head slightly elevated and fixed into the Mayfield clamp. An almost bicoronary skin incision was used to widely expose the patient's right frontal and parietal bone. After mobilization of the skin, the exophytic tumor mass was clearly identified ( Fig. 1C ). Using the 3D-printed craniotomy template, which was previously draped in a sterile film, the outline of the planned craniotomy was transmitted to the patient's skull surface. Afterward, the craniotomy was performed in a typical way. The bone flap and tumor were resected en bloc. As the dura was macroscopically invaded by the tumor, it was resected by a circular cut along the craniotomy outline ( Fig. 1C ). An alloplastic patch was used for dural closure. Then, the 3D-printed cranioplasty mold was also draped in a sterile film ( Fig. 2E ). The bottom of the mold was additionally covered in fat gauze. Now two component bone cement was mixed and filled into the mold. The cranioplasty implant was then formed under moderate pressure using the upper part of the mold ( Fig. 2F ). After the implant had cooled down, it was extracted from the mold and prepared for implantation ( Fig. 1G ). Multiple titanium plates were used for fixation. For drainage of potential epidural fluid collections, the implant was perforated several times.

### Manufacturing of the 3D-Printed Craniotomy Template and Cranioplasty Mold

3D Slicer was used to produce a virtual 3D model of the patient's skull based on thin-slice CT data. Afterward, the dataset was transferred to Design X (Oqton, Los Angeles, California, United States) for further editing. A virtual craniotomy with a safety margin of 10 mm was performed around the depicted lesion ( Fig. 2B ). The outline of the virtual craniotomy was then used to design a 3D-printed craniotomy template. The virtually excised bone was rejected for further planning of the cranioplasty. Using the Mesh Fit operation, the inner and outer surface of the skull were extrapolated for anatomical reconstruction of the bone defect. The Mesh Fit operation generates NURBS (Non-Uniform Rational B-Splines) surfaces through the implementation of an algorithm predicated on a mesh fitting paradigm. Based on the virtual cranial reconstruction a 3D-printed mold was constructed. The mold was capable of fabricating a cranioplasty of the exact size as the virtual craniotomy outline. Both the mold and template were then 3D printed using a PLA (Ultimaker, Geldermalsen, The Netherlands) on an Ultimaker 3 (Ultimaker, Geldermalsen, The Netherlands) FDM 3D printer ( Fig. 2C, D ).

### Histological Findings

**Fig. 3:**
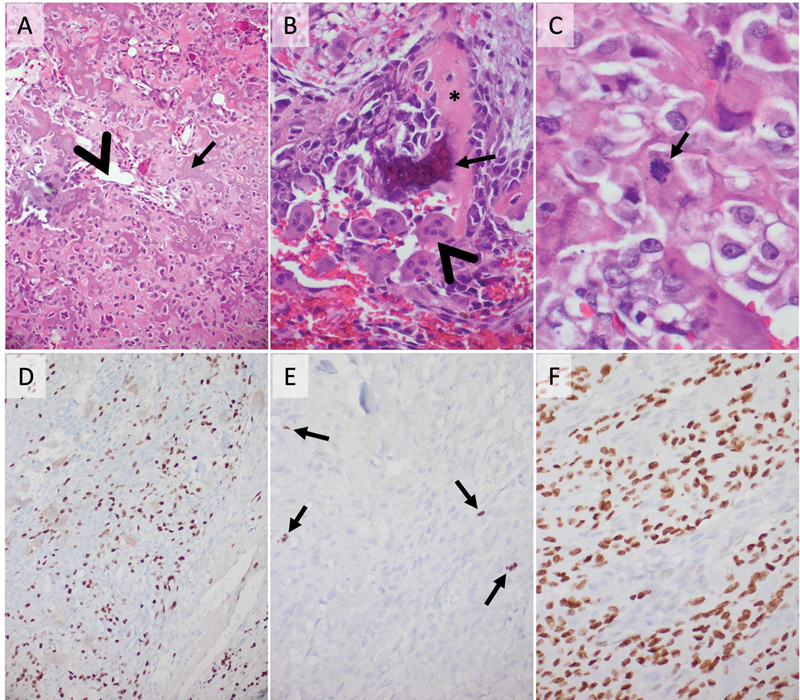
Morphological appearance of the tumor. ( **A** ) Hematoxylin and eosin (H&E) staining—a solid growing tumor, forming osteoid (arrow) with partially sinusoidally dilated blood vessels with a single layer of endothelial cells (arrowhead). ( **B** ) H&E staining—the osteoid (star) shows signs of calcification in certain regions (arrow) and multinucleated giant cells can be observed (arrowhead). ( **C** ) H&E staining—mitotic figures frequently appear (arrow). ( **D** ) K _i_ -67 immunohistochemistry—a focal proliferation rate of 50%. ( **E** ) PHH3 immunohistochemistry—up to four mitoses are seen in one high-power field (arrow). ( **F** ) SATB2 immunohistochemistry—positive labeling of tumor cell nuclei.

Microscopically, larger portions of regressively altered local bone with a fibrotically remodeled bone marrow were found. In focal areas, the bone with its bone ridges appeared narrowed, and then a fibrovascular stroma was seen within the enlarged bone marrow spaces. Partially growing into this bone in a cone shape along the marrow spaces, a pleomorphic tumor was observed, partially in larger areas, and partially only in small foci-forming osteoid ( Fig. 3A ). In some regions, the osteoid was calcified. There was a moderate increase in cell density. Cartilaginous tissue was not visible. The tumor cells were partly arranged in a trabecular pattern. Small vacuoles were found between the cells. The tumor cells often had an epithelioid configuration. Multinucleated giant cells occurred sporadically ( Fig. 3B ). Their cytoplasm appeared homogeneously eosinophilic to fine granular, sometimes also vesicular textured. The cells had mostly large, pleomorphic nuclei with moderately condensed, but sometimes also disaggregated fine granular chromatin. Prominent nucleoli were frequently evident. Mitotic figures were often seen, but altogether they showed no atypical configuration yet ( Fig. 3C ). Definite necrosis could not be identified, whereas structurally intact nuclei were only sporadically present in the context of the sometimes markedly increased osteoid. The intratumoral blood vessels emerged inconspicuous. Partly, they appeared sinusoidally dilated and had a single layer of endothelium. Using immunohistochemistry, the tumor showed a K _i_ -67 proliferation rate of 50% in some areas. In other regions, only 20% proliferating nuclei were observed ( Fig. 3D ). Using a PHH3-specific antibody, six mitoses were seen in 10 high-power fields ( Fig. 3E ). In addition, the nuclei were labeled positively with antibodies against SATB2 ( Fig. 3F ). No rearrangement of the USP6 gene was detected by FISH and no H3F3A K27 or G34 mutations were found by pyrosequencing. As a result, an osteoblastic, partially telangiectatic osteosarcoma of grade 3 was diagnosed.

### Postoperative Course

The patient was presented to the interdisciplinary sarcoma center at the Hannover Medical School. He was planned to receive systemic chemotherapy according to the EURAMOS protocol. Prior to his first therapy, he was referred to a fertility center for semen cryopreservation. Due to recurrent infectious mucositis, the therapy was conducted with reduced dosage and extended therapy intervals. With respect to long-term toxicity, the patient suffered from fatigue, polyneuropathia, and renal insufficiency. At 18-month follow-up, an MRI scan of the head and a staging CT did not show any sign of local recurrence or metastatic spread.

## Discussion

Primary cranial osteosarcoma is a rare entity with only 321 cases presented in the literature. 4 Here we present the case of a 20-year-old male patient, who had undergone a one-time surgical intervention with complete tumor resection and cranioplasty based on 3D-printed molds. The use of 3D-printed casting molds for the fabrication of tailored cranioplasties made from PMMA has already been described in the literature. 6 7 8 9 The combination of preoperative virtual planning with the aid of neuronavigation and fabrication of a corresponding PMMA mold for single-step surgery was first described by Anchieta. 10 The technique was further developed by da Silva Junior et al who produced 3D-printed templates for single-step frame-guided resection and cranioplasty for surgery of intraosseous lesions. 9 However, to our knowledge this is the first presentation of a tailored craniotomy template and corresponding molds for cranioplasty enabling single-session surgery for osteosarcoma of the skull. The process from designing to manufacturing of the implant took 1 week. Close coordination between engineers and surgeons was necessary during this phase. The material costs for the required amount of 200 g PLA for 3D printing of the mold and template amount to 8€, assuming a price of approximately 40 €/kg PLA. In addition, we calculated 150€ for the amount of bone cement used in the presented case. The costs for the purchase and operation of the 3D printer as well as personnel costs were not taken into account, as the implant was manufactured by our interdisciplinary research team to patient-specific demand. Nevertheless, da Silva Júnior et al recently highlighted the cost-effectiveness of the method. In their case series, the cost of the implant was reduced by more than half compared with conventional implants. 9 The craniotomy template allowed en bloc resection of the tumor with a safety margin of at least 10 mm on the entire circumference. The coronal suture and the convexity of the skull served as markers for orientation of the template. 9 As cranial osteosarcomas tend to local recurrence, a tumor-free resection margin and excision of infiltrated dura is of crucial importance for further treatment and prognosis. 11 12 The corresponding molds then facilitated fabrication of an PMMA implant of the exact size of the craniotomy defect. The size and shape of the implant led to a very satisfactory cosmetic result ( Fig. 1E,F,H ). Worth mentioning is that the 3D-printed template and molds did not get in contact with human tissue. They were covered in a sterile film and the actual implant was made of PMMA, which has been used for free-hand cranioplasty over decades. In contrast to the molding technique, intraoperative free-hand fabrication of these implants requires a good 3D imagination to produce good cosmetic results. Although Fischer et al reported poor results in less than 10% of all PMMA cranioplasties in their series, the aesthetic outcome is often not considered and the use of computer-aided design techniques is strongly recommended in large cranioplasties. 13 14 According to the Medical Device Regulation of the European Union, we consider our implant to be a needs-adapted, custom-made product. Although an uneventful course is presented, the routine application of these technologies is limited by a lack of certification of the fabrication process. Usually, neoadjuvant chemotherapy precedes surgery in the treatment pathway of osteosarcomas. 15 However, in the presented case, the rapid progression and intracranial midline shift required prompt surgical resection. Moreover, based on the patient's history, malignancy primarily wasn't considered. Our surgical strategy then allowed us to start the high-dose chemotherapy treatment immediately after wound healing without the need to plan additional surgery for cranial reconstruction. Considering the prognostic impact of a delay or interruption of the chemotherapy, this could have had a beneficial effect on the patients' treatment. 16 The patient received adjuvant chemotherapy according to the EURAMOS protocol. Despite the need to reduce the dosage and therapy interval due to recurrent infectious mucositis, he did not show any signs of local tumor recurrence or metastatic disease on imaging at 18-month follow-up. He still suffered from fatigue, polyneuropathy, and renal insufficiency, which all are typical side effects of the polychemotherapy. As the patient has recognized a right frontal surface irregularity for almost 10 years by the time of diagnosis, malignant transformation of a former benign lesion, as it has previously been described in the literature, is possible. 17 This emphasizes the need for early diagnosis confirmation from histopathological examination and to perform complete resection of supposedly benign bone neoplasms in young patients whenever possible, especially if a previously asymptomatic lesion becomes symptomatic.

## Conclusion

Primary cranial osteosarcoma is a rare condition, which, given the absence of evidence based on statistics, needs a custom-made surgical approach in all cases. The need to perform a bony resection with tumor-free margins can create technical difficulties. Furthermore, the need to perform reconstructive surgery can cause undue delays in the further oncological treatments. Our case shows that the use of 3D-printed molds to facilitate a safe preoperative planning of the resection area, and a single-session surgery including a custom-made cranioplasty responding to the highest esthetical standards, can resolve both problems in a satisfactory manner.
